# The WHO Hub for Pandemic and Epidemic Intelligence; supporting better preparedness for future health emergencies

**DOI:** 10.2807/1560-7917.ES.2022.27.20.2200385

**Published:** 2022-05-19

**Authors:** Oliver Morgan, Richard Pebody

**Affiliations:** 1 World Health Organization (WHO) Hub for Pandemic and Epidemic Intelligence, Berlin, Germany; 2 World Health Organization (WHO) Regional Office for Europe, Copenhagen, Denmark

**Keywords:** public health intelligence, public health Europe, outbreaks, public health policy, surveillance

Since 2016, when the World Health Organization (WHO) Health Emergencies Programme was established, WHO has transformed its capabilities to reliably prepare for, detect, and respond to emerging threats and humanitarian crises around the world. Every month, WHO identifies 4,500 signals of public health events and in 2020, WHO responded to 116 emergencies in 194 countries, territories, and areas [1]. The recent coronavirus disease (COVID-19) pandemic has exemplified that surveillance of emerging threats remains challenging, owing to a number of limitations, including data fragmentation and information systems that are not interoperable, limited integration of health information with other contextual information, such as climate information and social factors, little standardisation of analytical tools and methods, difficulties in assessing the effectiveness of public health interventions in real time, sub-optimal use of data-driven insights for public health decision-making [2] and, importantly, a weak global trust architecture for data and information sharing [3].

The WHO Hub for Pandemic and Epidemic Intelligence, established on 1 September 2021 with EUR 90 million funding from the Federal Republic of Germany, aims to transform the global surveillance of emerging public health threats and to build a collaborative intelligence ecosystem (Box). It forms part of a new Division of Health Emergency Intelligence and Surveillance Systems, which is located within the WHO Headquarters Health Emergencies Programme. The Division brings together many aspects of the organisation’s work in surveillance for emergencies and works with all Member States and partners along with the six WHO Regional Offices; this includes the WHO Regional Office for Europe (WHO/Europe) and its 53 Member States, the European Commission and other institutions with many of whom WHO has long-standing collaborations.

Box **Strategic objectives of the WHO Hub for Pandemic and Epidemic Intelligence**

**Connect**

• ***Create a multi-disciplinary collaborative intelligence environment***

Build trust-based collaborations for pandemic and epidemic intelligence across sectors and disciplines.

• ***Build a global system to connect data from a wide range of sources***

Lead the development of a global system that promotes greater accessibility and usability of diverse data while preserving data custodianship with data owners

**Innovate**

• ***Facilitate the development and wide availability of robust analytic tools***

Build open processes that facilitate experimentation, testing, and scaling of innovations in data analytics, including modelling and genomic surveillance.

• ***Drive a global agenda for responsible R*** & ***D agenda for pandemic and epidemic intelligence***

Establish research priorities for practitioners, academic institutions, and funders, and facilitate the translation of research into practice.

**Strengthen**

• ***Provide advice, training, and capacity-building services***

Provide technical guidance and training, with national public health institutes and other partners to scale up efforts, to strengthen countries’ pandemic and epidemic intelligence capacities.

• ***Support timely, effective decision-making and policies***

Strengthen decision-making through greater sharing of insights and collaborative problem solving through building a robust trust architecture and data solidarity.

R&D: Research and Development; WHO: World Health Organization.

In the context of future collaboration with our partners, the WHO Pandemic Hub and WHO/Europe welcome the strengthening of the ECDC mandate, especially greater capabilities for data analytics; the development of digital platforms and a wider range of data sources; greater foresight for risk management; and a greater focus on regional and global health threats beyond countries in the European Union/European Economic Area [4]. The Pandemic Hub also recognises the potential role of ECDC’s proposed European Health Task Force for serious cross-border health threats to improve access to scientific data, analytic capabilities, and decision-support tools [5].

The WHO Hub also collaborates with the newly established Health Emergency Preparedness and Response Authority (HERA), which aims to improve the European Union’s capacity to respond to cross-border health threats and to employ medical countermeasures [6]. The two bodies are exploring collaborative intelligence approaches to sharing information and developing common digital capabilities. An ongoing collaboration of the Pandemic Hub with the Joint Research Centre of the European Commission is developing the Epidemic Intelligence from Open Sources (EIOS) platform [7]. EIOS currently used by ECDC and 43 WHO Member States worldwide, uses artificial intelligence to identify signals of public health threats from more than 35,000 data feeds.

Developing analytic tools and capabilities for genomic data and strengthening bioinformatics is of crucial importance in pandemic preparedness and the Pandemic Hub will host the International Pathogen Surveillance Network, an initiative to strengthen global genomic surveillance for emerging pathogens [8,9]. WHO recently hosted a consultation meeting to establish principles for the global sharing of such data, working with, among others, the Research and Innovation Commission [10] and the European Molecular Biology Laboratory’s European Bioinformatics Institute. Initiatives funded by the European Union’s Horizon Europe programme are creating opportunities to build a collaborative intelligence ecosystem within Europe, such as the Versatile Emerging infectious disease Observatory and Monitoring Outbreak events for Disease surveillance, in a data science context [11,12]. At the strategic level, the Pandemic Hub also collaborates with European Commission Directorates such as DG SANTE, DG INTPA and DG CONNECT.

The German national public health institute, the Robert Koch Institute in Berlin and the Pandemic Hub have a joint work plan that includes genomic surveillance, surveillance for antimicrobial-resistant organisms, strengthening epidemic intelligence capabilities globally, and technical exchange with institutions in low- and middle-income countries, in collaboration with the Global Outbreak and Alert Network (GOARN). With the International Association of National Institute of Public Health Institutes, based in France, RKI and the WHO Pandemic Hub are assessing the surveillance needs of national public health institutes within Europe and other regions of the world.

Creating a global surveillance ecosystem with interoperable data and information platforms is high on the WHO Pandemic Hub’s agenda and it thus also works with different initiatives beyond Europe. A project within the Health Security Partnership of the Global Partnership for Biosecurity to Strengthen Surveillance in Africa will be launched in summer 2022; it builds collaborations between Africa Centers for Disease Control (CDC), the Robert Koch Institute, the WHO Regional Office for Africa, and the WHO Regional Office for the Eastern Mediterranean. The Pandemic Hub further collaborates with the United States (US) CDC’s Center for Forecasting and Analytics. The Center is a large investment to strengthen public health surveillance and forecasting in the US and brings together 50 international modelling groups to provide support to decisionmakers at global, national, and local levels [13]. The WHO Pandemic Hub is also collaborating with global initiatives by philanthropic organisations such as the Rockefeller Pandemic Prevention Institute, the Wellcome Trust, and the Bill and Melinda Gates Foundation [14].

Academic institutions are important actors in the pandemic and epidemic intelligence ecosystem. During the COVID-19 pandemic, they have much advanced analytics and modelling, worked with WHO and other national and international public health institutions to support recommendations on vaccine policy as well as the implementation of public health and social measures, and contributed to government decision-making, for example, through the United Kingdom’s Scientific Pandemic Influenza Group on Modelling [15]. Another notable example is the Network for Genomic Surveillance in South Africa, a collaboration of academic and public sector laboratories established in 2020 in response to the COVID-19 pandemic.

Engagement between academic institutions and government decisionmakers for public health remains largely ad hoc. It is still unclear how well research investments in pandemic and epidemic intelligence are aligned globally with the priorities of operational public health agencies. The WHO Pandemic Hub is developing an R&D agenda for pandemic and epidemic intelligence that will support researchers, commissioners of research, and public health institutions with prioritisation and enable collaboration between countries and regions. The Pandemic Hub is building its R&D agenda in collaboration with the Charité University in Berlin, Germany as well as with the Karolinska Institute in Stockholm, Sweden and with the Global Research Collaboration for Infectious Disease Preparedness.

The WHO Pandemic Hub is a catalytic initiative to build a global surveillance ecosystem through collaboration for improved pandemic and epidemic intelligence. Across the entire-WHO European Region there are considerable capabilities and strong existing collaborative networks. At the same time, the European Union is making formidable investments that are providing important momentum for technical and financial investment for public health surveillance. These and other investments will achieve their maximum potential in collaboration with both other countries in the WHO European Region as well as globally [16]. The Pandemic Hub is designed to foster strategic investments by promoting collaborative approaches and supporting prioritisation. No single institution or initiative will be able to make the world better prepared for the next pandemic. We will be prepared only by ensuring that all countries and communities are included in preparedness activities. The World Health Organization is committed to this vision; the contributions of the whole of the WHO European Region will be crucial for our collaborative success.
